# Oral Lesions and Oral Health-Related Quality of Life in Adult Patients with Psoriasis: A Retrospective Chart Review

**DOI:** 10.3390/life14030347

**Published:** 2024-03-07

**Authors:** Federica Di Spirito, Annunziata Raimondo, Maria Pia Di Palo, Stefano Martina, Mario Fordellone, Donato Rosa, Massimo Amato, Serena Lembo

**Affiliations:** 1 Department of Medicine, Surgery and Dentistry, University of Salerno, 84084 Baronissi, Italy; araimondo@unisa.it (A.R.); mariapia140497@gmail.com (M.P.D.P.); drosa@unisa.it (D.R.); mamato@unisa.it (M.A.); slembo@unisa.it (S.L.); 2 Department of Mental, Physical Health and Preventive Medicine, Università degli Studi della Campania “Luigi Vanvitelli”, 80138 Napoli, Italy; mario.fordellone@unicampania.it

**Keywords:** psoriasis, skin diseases, quality of life, oral lesions, mouth, oral health, oral manifestations

## Abstract

Psoriasis is a widespread chronic inflammatory skin disease, that negatively affects physical and emotional well-being and quality of life, as shown by the generally low Dermatology Life Quality Index (DLQI). Psoriasis is burdened by associated comorbidities and some patients manifest concurrent oral lesions, although the existence of oral psoriasis remains controversial. Psoriasis-specific and nonspecific oral lesions and Oral Health-Related Quality of Life (OHRQoL), self-assessed using the Oral Health Impact Profile-14 (OHIP-14) questionnaire, were retrospectively reviewed in adult untreated psoriasis patients with ≥15 teeth, who were non-smokers and had no dental or periodontal infections. Sample (age, gender, comorbidities) and descriptive variables (Body Surface Area-BSA, Psoriasis Area and Severity Index-PASI, Dermatology Life Quality Index-DLQI, severity of psoriasis, distribution of lesions and predominant involvement, years since diagnosis) were correlated with DLQI and OHIP-14 and compared by baseline DLQI and OHRQoL classes. Charts from 90 participants were included. No oral lesions were detected, and excellent/good OHRQoL was found in 94% of the participants. DLQI scores displayed positive significant associations with PASI and BSA, while OHIP-14 with hypertension and IMID, and age. PASI and BSA were significantly higher in participants with DLQI > 10 and also differed significantly among OHQRoL ranks, as well as mucosal involvement and comorbidities. Specifically, among subjects revealing an Excellent OHQRoL, 92.6% were non-IMID, 75% non-hypertensive, 89.7% non-diabetic subjects, 86.8% of non CVD-subjects.

## 1. Introduction

Psoriasis is a persistent inflammatory skin disease characterized by inflammation affecting both the dermis and the epidermis [1], with a prevalence ranging from 2% to 4% in the general population [2]. The etiology of the disease still needs to be clearly understood. However, epidemiological studies have identified various risk factors, including smoking, a high body mass index, lack of exercise, excessive alcohol consumption, and genetic predisposition [2].

Psoriasis is now recognized as a systemic disease [3]. Numerous comorbidities exacerbate the burden of psoriasis [4]. People who have psoriasis have an increased susceptibility to developing other severe and chronic conditions, such as psoriatic arthritis, metabolic syndrome or its components, cardiovascular disease, anxiety, depression, and Crohn’s disease [5]. In addition, psoriasis has a notable impact on physical and emotional well-being, which is associated with quality of life (QoL) [6].

Among the outcome parameters predominantly used to assess the impact of psoriasis on QoL are the Dermatology Life Quality Index (DLQI) and the Skindex-29 [7,8]. The DLQI, a widely used scale, has been translated into all languages represented in the consensus program and is freely available online for academic and office use [7,9].

Certain individuals with psoriasis exhibit synchronous oral lesions in addition to their skin disease. Although the existence of oral psoriasis remains controversial, involvement of the oral mucosa and other mucous membranes does occur, albeit rarely [10,11]. In any case, patients with psoriasis had a higher incidence of abnormal oral mucosa (74%) compared to the general population (46%) [12]. Nevertheless, oral psoriasis lesions have no consistent pattern [13,14]. Lesions in the oral cavity of people with psoriasis can be divided into psoriasis-specific lesions and nonspecific lesions, which can occur independently of psoriasis and during other diseases.

Psoriasis-specific oral lesions usually co-occur with skin involvement, although cases have been reported in which oral psoriasis precedes the development of skin lesions. These mucosal lesions have microscopic features similar to skin psoriasis, although their clinical appearance is very heterogeneous, and manifest as white or gray plaques, ring-shaped lesions, diffuse areas of erythema, edema, acute inflammatory infiltrates of the epithelium, mixed infiltrates of the lamina propria with neutrophils and lymphocytes, and organized neutrophilic microabscesses. Oral scarring may occur in some individuals with mucosal psoriasis [10]. Oral lesions do not have a uniform pattern and may have different appearances [10]. Therefore, isolated reports of oral lesions with characteristic histologic changes in the absence of psoriasis of the skin may be manifestations of psoriasis in patients in remission from previous skin disease or patients with a positive family history. Without the above diagnostic criteria, suspicious oral findings may be considered psoriasiform mucositis and not oral psoriasis [15].

Nonspecific lesions of the oral mucosa of psoriasis patients mainly include a fissured tongue, which proves to be the most frequently documented oral abnormality associated with psoriasis, followed by geographic tongue and other oral manifestations whose association is not clear, such as cheilitis, leukokeratosis, erythematous macules, fibromas, and depapillation of the tongue [12].

A comprehensive assessment of oral health in psoriasis patients requires the inclusion of patient self-reports of oral health, as patients’ and healthcare professionals’ perceptions are unlikely to be consistent [16,17]. Integrating both subjective and objective health assessments is critical for refining health promotion strategies, disease prevention programs, and equitable distribution of health resources [18]. As part of this effort, numerous measures of oral health-related quality of life (OHRQoL) have been developed [19,20,21,22]. Some of these assessments aim to provide an index of the impact of oral health disorders, while others focus on functional disorders and their associated social consequences.

A shortened version of the original Oral Health Impact Profile (OHIP-49) derived from the original profile and known as OHIP-14, is a 14-item questionnaire that has been developed and validated [17,23]. Although the authors concede that the shortening of the scale entails certain reductions in the scope of the original OHIP scale, the practical utility of OHIP-14 was increased. Its use in cross-sectional surveys is documented, and its discriminant validity is well established [17,24].

While the existing body of literature explored the ramifications of diverse health conditions on OHRQoL, a noticeable dearth of information persists concerning the precise influence of psoriasis on OHRQoL. Furthermore, an evident research gap exists, as no studies have endeavored to establish correlations between the impact of oral alterations, as assessed by the OHIP-14, and its interconnectedness with QoL of adult patients suffering from psoriasis measured by the DLQI.

Therefore, the present retrospective chart review aimed to estimate the prevalence of psoriasis-specific and nonspecific oral lesions and outline the self-reported OHRQoL using the Oral Health Impact Profile-14 (OHIP-14) questionnaire in adult untreated psoriasis subjects with ≥15 teeth, no smoking habits, and no oral mucosal, dental or periodontal infections.

The secondary objectives were to evaluate the correlations between perceived self-reported quality of life related to the skin (DLQI scores) and oral (OHIP-14 scores) conditions with the sample characteristics (age, gender, comorbidities) and psoriasis descriptive variables (Body Surface Area—BSA, Psoriasis Area and Severity Index—PASI, Dermatology Life Quality Index—DLQI, severity of psoriasis, distribution of lesions and predominant involvement, years since diagnosis), and to compare all variables in relation to gender, DLQI, years since diagnosis and comorbidities.

## 2. Materials and Methods

### 2.1. Study Design and Sample

Paper-based and electronic medical and dental records, including a comprehensive medical history, drug use, and psoriasis diagnosis, characteristics, and therapy, as well as outcomes of the oral clinical examination, of eligible psoriasis individuals attending the dermatologic unit of Azienda Ospedaliero-Universitaria San Giovanni di Dio e Ruggi d’Aragona, Salerno, Italy, from June 2022 to May 2023, were retrospectively reviewed.

The research adhered to the ethical guidelines outlined in the Declaration of Helsinki regarding experimentation involving human subjects. Approval for the study was obtained from the local Ethical Committee (University of Salerno), with the endorsement provided through Protocol No. 51/2022 approved on 16 February 2022.

Before their participation, all subjects provided written informed consent, emphasizing the ethical consideration and voluntary nature of their involvement in the study. All patients whose charts were included in the study underwent the standard diagnostic and treatment protocol.

The sample size was obtained from previous studies [25].

#### 2.1.1. Inclusion Criteria

❖ Age ≥ 18;
❖ Definitive diagnosis of Psoriasis;
❖ Not under treatment for psoriasis (for at least 1 year);
❖ ≥15 teeth.

#### 2.1.2. Exclusion Criteria

❖ Pregnant and lactating women;
❖ Current or previous neoplasm, chemotherapy, radiotherapy, medication-related osteonecrosis of the jaws;
❖ Smoking habit;
❖ dental and periodontal infections requiring dental treatment;
❖ Removable dentures.

### 2.2. Data Collection

Data from subjects compliant with the eligibility criteria detailed below were electronically retrieved from medical and dental charts and recorded for automatic computations.

#### 2.2.1. Sample Characteristics

The following descriptive variables were recorded from participants’ records:

✓ Age;
✓ Gender;
✓ Comorbidities: a positive medical history for type 2 diabetes, hypertension, dyslipidemia, cardiovascular events, or immune-mediated inflammatory diseases (IMIDs), as well as habitual drug use.

#### 2.2.2. Psoriasis Descriptive Variables

▪ Psoriasis subtypes: diverse clinical forms encompass chronic plaque psoriasis, identifiable by well-demarcated, erythematous plaques covered with coarse scales; guttate psoriasis, characterized by the sudden onset of numerous small inflammatory plaques; pustular psoriasis, presenting as an acute, subacute, or chronic pustular eruption, and erythrodermic psoriasis, featuring widespread cutaneous erythema and scaling affecting a substantial portion or the entirety of the body surface area [11].
▪ Body Surface Area (BSA): delineated as the percentage of total body surface involvement, with 1% representing an approximate area equivalent to the patient’s handprint.
▪ Psoriasis Area and Severity Index (PASI): serves as a widely employed tool in psoriasis trials, offering an assessment and grading of the severity of psoriasis lesions along with the patient’s response to treatment. It generates a numeric score within the range of 0 to 72. Typically, a PASI score falling between 5 and 10 indicates moderate disease, while a score exceeding 10 is considered severe. The benchmark for efficacy in most clinical trials and the criterion endorsed by the Food and Drug Administration for evaluating new psoriasis treatments is a 75% reduction in the PASI score, commonly referred to as PASI 75 [7].
▪ Dermatology Life Quality Index (DLQI): is a validated patient-reported instrument designed to assess the impact of skin diseases on health-related quality of life and daily activities. Comprising 10 questions, each response is evaluated on a scale from 0 to 3. The DLQI score is calculated by summing the scores for each question, yielding a maximum score of 30 and a minimum score of 0. Higher scores on the DLQI indicate a greater compromise in the quality of life, with a score exceeding 10 suggesting a severe impact on the patient’s life due to their skin condition [7]. In detail, DLQI score categorized the impact of the disease on the quality of life into none (0–1 score), small (2–5 score), moderate (6–10 score), very large (11–20 score), and extremely large (21–30 score) [3]. Developed by Finlay AY and Khan GK in 1994, the DLQI has been widely utilized in global clinical trials and research endeavors exploring the quality of life and disease burden associated with various dermatological conditions. Accessible in multiple languages representative of the participating countries, the DLQI can be accessed online (http://www.dermatology.org.uk/quality/quality-dlqi.html, accessed on 20 November 2023). The questionnaire was completed as part of a standardized interview to avoid different interpretations among patients and to mitigate its subjective nature [26] and was electronically attached to the patients’ medical records.
▪ Psoriasis severity: mild psoriasis was characterized by the criteria of body surface area (BSA) ≤ 10, psoriasis area and severity index (PASI) ≤ 10, and dermatology life quality index (DLQI) ≤ 10; conversely, moderate to severe psoriasis was defined as (BSA > 10 or PASI > 10) and DLQI > 10.

It is noteworthy that specific clinical scenarios, such as the involvement of visible areas or severe nail involvement, may alter the classification of psoriasis from mild to moderate to severe [27].

▪ Psoriasis lesions distribution and prevailing involvement (skin, mucosal, nail involvement, and diffuse distribution of lesions);
▪ Psoriasis arthritis;
▪ Years since psoriasis diagnosis.

#### 2.2.3. Outcome Variables

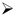

Oral lesions: psoriasis-specific lesions and nonspecific lesions of the oral mucosa reported on dental charts, along with mucosal, dental, and periodontal infections and treatment needs, were obtained from dental charts granted by the Complex Operating Unit of Odontostomatology of the same Hospital.

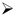

Oral Health Impact Profile-14 (OHIP-14): scores have a potential range from 0 to 56, with the calculation involving the summation of the ordinal values assigned to each of the 14 items. Additionally, the domain scores within this instrument can span from 0 to 8. A higher OHIP-14 score is indicative of a more compromised Oral Health-Related Quality of Life (OHRQoL), while lower scores suggest a better OHRQoL [28]. The Italian-validated version of the questionnaire (provided as Supplementary File S1) [29], obtained from dental charts, was completed as part of a standardized interview to avoid different interpretations among patients and to mitigate its subjective nature [26]. To make the results more readable, an OHIP score of 0–14 was associated with excellent OHRQoL, an OHIP score of 15–28 with good OHRQoL, an OHIP score of 27–42 with medium OHRQoL and an OHIP score of 43–56 with low OHRQoL.

### 2.3. Data Analysis

Continuous variables were reported as either means and standard deviation or median and interquartile ranges (IQRs) according to their distribution, as assessed by the Shapiro–Wilk normality test. Categorical variables were reported as absolute frequencies and percentages.

Three different linear regression models were performed for DLQI and OHIP14 scores (i.e., response variables) using PASI and BSA as principal covariates. The covariates for adjusting in the first model were age, gender, and years since psoriasis diagnosis; for the second model, all the comorbidities were used; finally, in the last model, the psoriatic lesion variables were used.

Differences in baseline characteristics of DLQI classes were tested by t-student or Wilcoxon tests (according to their distribution) for continuous variables and Pearson chi-squared or Fisher’s exact tests for categorical variables. Differences in baseline characteristics of OHRQoL classes were tested by ANOVA or Kruskal–Wallis tests (according to their distribution) for continuous, and Pearson chi-squared or Fisher’s exact tests for categorical variables.

All the statistical analyses were performed with the R Studio Statistical software, version 4.1.3. Statistical tests with *p*-values smaller than 0.05 were considered statistically significant.

## 3. Results

Data from medical and dental charts of 90 participants meeting the eligibility criteria were included in the study.

### 3.1. Qualitative Synthesis of the Collected Data

#### 3.1.1. Sample Characteristics

Psoriasis patients participating in the study were 56% males and 44% females between 18 and 77 years of age (mean: 52.1; median 53.0; standard deviation: 14.1).

**Figure 1:**
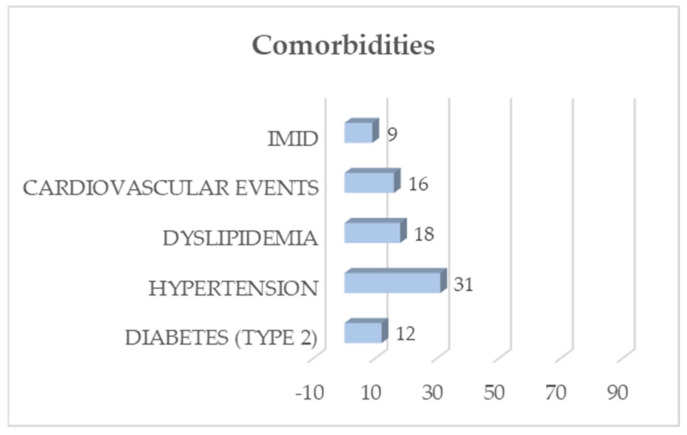
Participants’ comorbidities on the x axis, the comorbidities investigated (diabetes, hypertension, dyslipidemia, cardiovascular events, and IMID); on the y axis, the number of participants.

Among these, 43 subjects reported neither comorbidities nor habitual drug use, while 47 participants had a positive medical history (Figure 1) for type 2 diabetes, hypertension, dyslipidemia, cardiovascular events, or IMIDs, either individually or in combination, as illustrated in Figure 1.

#### 3.1.2. Psoriasis Descriptive Variables

Seventy-four participants were diagnosed with Chronic Plaque psoriasis, while nine subjects were diagnosed with Pustulosa, five with Guttate, and two with Erythrodermic subtypes.

Twenty-six participants (approximately 30%) suffered from Psoriasis arthritis, while 64 (approximately 70%) did not.

**Figure 2:**
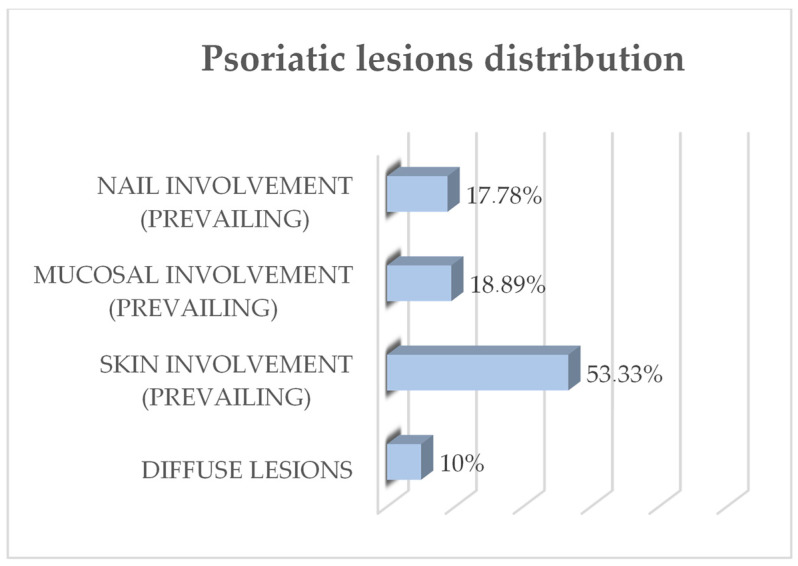
Participants’ psoriasis lesions distribution on the body and prevailing involvement.

Regarding the distribution of psoriasis lesions, 48 patients exhibited prevailing skin involvement, 17 mucosal (mainly genital) involvement, 16 nail involvement, and nine diffuse distribution of lesions (Figure 2).

The mean Body Surface Area (BSA) was 23.5, while the mean PASI was 8.63 (median: 6, standard deviation: 9.16), and the mean DLQI score was 12.8 (median: 12, standard deviation: 8.59).

Health-related quality of life (HRQoL) was assessed using the Dermatology Life Quality Index (DLQI), with 43 subjects scoring ≤ 10 and 47 scoring > 10.

Psoriasis severity, assessed as described above, revealed mild psoriasis in 21% of cases and moderate to severe psoriasis in 79% of cases.

The mean duration since the diagnosis of psoriasis was 16.2 years (minimum: 1, maximum: 60; median: 11.0; standard deviation: 13.8 years). Specifically, 48% of cases were diagnosed within 10 years of psoriasis diagnosis, while 52% were diagnosed more than 10 years ago.

#### 3.1.3. Outcome Variables: Oral Lesions and OHQRoL in Psoriasis Subjects

No psoriasis-specific lesions and nonspecific lesions of the oral mucosa were reported on dental charts.

The Oral Health Impact Profile (OHIP) questionnaire, evaluating the Oral health-related quality of life (OHRQoL), showed a 100% completion rate. OHIP-14 scores ranged from 0 to 52 (mean: 8.58; median: 3.00; standard deviation: 11.6).

Sixty-seven participants reported excellent OHRQoL (OHIP score 0–14), 18 good OHRQoL (OHIP score 15–28), three medium OHRQoL (OHIP score 27–42), and two low OHRQoL (OHIP score 43–56).

### 3.2. Variables Correlations with DLQI and OHIP-14 Scores

DLQI scores exhibited positive significant associations with both PASI and BSA scores., as displayed in Table 1.

In the context of Oral Health-Related Quality of Life, OHIP-14 scores displayed positive significant associations with a medical history of hypertension (*p*-value < 0.001), IMID (*p*-value = 0.007), and age (*p*-value = 0.004), as shown in Table 2.

### 3.3. Variables Differences Related to DLQI and OHIP

PASI (*p*-value = 0.00) and BSA (*p*-value = 0.00) values differed significantly between subjects with DLQI ≤ 10 and DLQI > 10 (Table 3).

**Figure 3:**
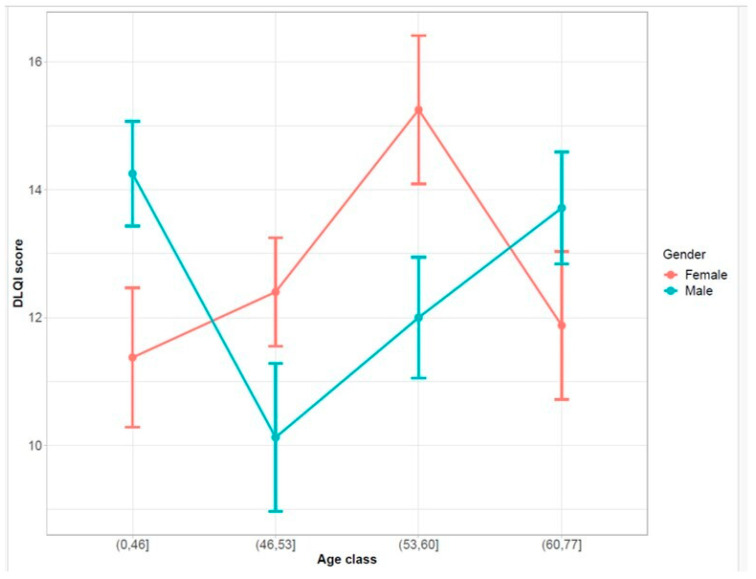
DLQI trend by age classes was stratified for males and females. For the construction of these plots, the age variable was categorized in four classes divided by quartiles: the point represents the mean of DLQI score for both gender and age class, and the upper/lower band represents the confidence interval at 95%.

The DLQI trend by age classes was stratified for males and females (Figure 3), showing that in the extreme age classes (i.e., 0–46 and 60–77) the DLQI scores of males were higher than those of females. Moreover, in all the age classes, the differences between males and females are statistically significant, except for the 60–77 class.

PASI (*p*-value = 0.01), BSA (*p*-value = 0.11), mucosal (mainly genital) involvement (*p*-value = 0.03), and comorbidities differed significantly among OHQRoL ranks.

Specifically, among subjects revealing an Excellent OHQRoL (OHIP score of 0–14), 92.6% were non-IMID (*p*-value = 0.02), 75% non-hypertensive (*p*-value = 0.00), 89.7% non-diabetic subjects (*p*-value = 0.02), 86.8% of non CVD-subjects (*p*-value = 0.01) (Table 4).

**Figure 4:**
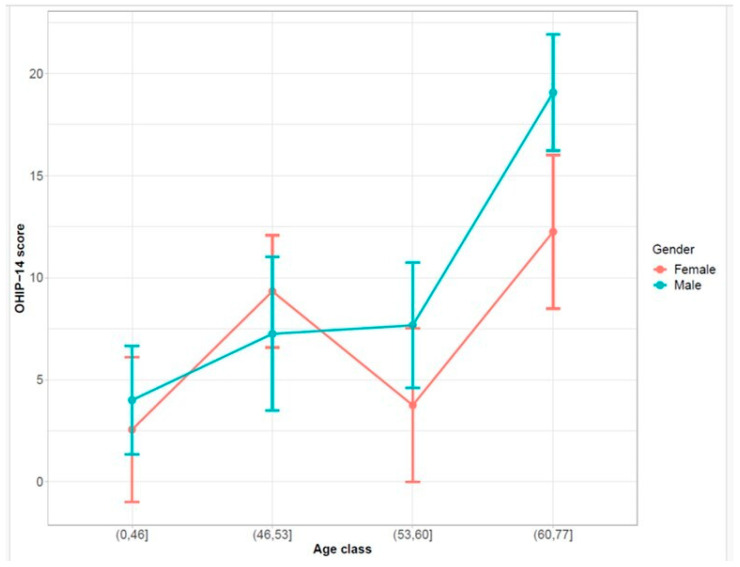
DLQI trend by age classes was stratified for males and females. For the construction of these plots, the age variable was categorized into four classes divided by quartiles: the point represents the mean of OHIP-14 score for both gender and age class, and the upper/lower band represents the confidence interval at 95%.

The OHIP-14 trend by age classes was stratified for males and females (Figure 4), the last age class (i.e., 60–77) OHIP-14 score is higher than the other classes for both males and females. Moreover, in all the age classes, the differences between males and females are not statistically significant.

## 4. Discussion

The primary aim of the study was to estimate the prevalence of psoriasis-specific and nonspecific oral lesions retrospectively and to outline the Oral Health-Related Quality of Life (OHRQoL), determined through the self-administered Oral Health Impact Profile-14 (OHIP-14) questionnaire, in adult untreated psoriasis subjects with ≥15 teeth, no smoking habits, and no dental or periodontal infections.

### 4.1. Outcome Variables: Oral Lesions and OHQRoL in Psoriasis Subjects

#### 4.1.1. Psoriasis-Specific and Nonspecific Oral Lesions in Adult Untreated Psoriasis Subjects

Geographic tongue is the oral abnormality most extensively studied in the context of psoriasis, with its prevalence estimated to fall between 5% and 18% in psoriasis patients. The justification for its association lies in the similarity of its fundamental lesions at the microscopic level and the presence of a shared genetic marker, HLA-Cw6. Microtrauma resulting from activities like chewing and speaking on the tongue may contribute to the Koebner phenomenon, potentially stimulating the appearance of the geographic tongue [12]. However, no diagnosis of geographic tongue was recorded in the participants’ dental charts.

Instead, the prevalent alterations observed in psoriasis patients by Olejnik et al. included fissured tongue (approximately 40%), beyond white-coated tongue (approximately 25%), and linea alba (approximately 20%) [3]. Fissured tongue also emerged as the most prevalent abnormality observed in individuals with psoriasis in the cross-sectional study by Altemir et al. [12], showing an overall prevalence ranging from 14% to 47% among psoriasis subjects across various populations. Fissured tongue, also known as lingua fissurata, lingua plicata, scrotal tongue, or grooved tongue, is clinically recognizable by an anteroposterior groove, often accompanied by multiple lateral fissures. It appears to be persistent [10], and is considered a possible sequel of geographic tongue in psoriasis patients [10], with no significant difference in the occurrence between early and late-onset psoriasis. Its prevalence is typically higher among men and increases with age [10]. Nevertheless, although the mean age of our sample was 52.1 (median 53.0; standard deviation: 14.1), no cases diagnosed with fissured tongue were retrieved through the retrospective chart review, possibly because it is estimated to be more common in pustular psoriasis, which was presently described in only 10% of participants.

Denture-related stomatitis was also diagnosed in about 7% of participants [3]. Indeed, psoriasis-associated oral mucosal lesions are considered primarily localized on the tongue [3], along with a significantly higher incidence of angular cheilitis and denture-related stomatitis in psoriasis patients compared to the general population, as also found by Costa et al. [1]. The latter proposed that *Candida* spp. biofilm, a major contributor to *Candida*-associated denture stomatitis, can be influenced by subtherapeutic levels of polyenes persisting in the oral cavity after topical treatment during the ‘adherence phase’ [3], potentially justifying the absence of angular cheilitis and denture stomatitis in our untreated sample, not including subjects with active infections nor removable prosthesis.

In any case, beyond nonspecific lesions of the oral mucosa, there is no consensus on the psoriasis-specific lesions. Indeed, various morphological patterns have been delineated. These encompass diffuse, intense mucosal erythema linked with acute psoriasis flares, well-defined annular lesions in white or grayish-yellow hues, and mixed presentations involving ulcerative, vesicular, pustular, and indurated entities. Psoriasis manifestations may affect different oral locations, with the buccal mucosa being the most commonly involved. Unusual sites such as the palate and gingiva have been reported infrequently [15]. Additionally, pinpoint bleeding reminiscent of the cutaneous Auspitz’s sign may be observable in the affected mucosa [15]. Those oral psoriasis-specific lesions share similar histopathological features with their cutaneous counterparts and follow a clinical course parallel to the cutaneous disease. Nonetheless, no psoriasis-specific lesions of the oral mucosa have been retrieved from dental charts, even if 79% of participants had moderate to severe psoriasis.

Hence, contrary to Olejnik et al. recording oral mucosal lesions in 69% of the participants [3], and also reporting in certain cases, multiple types of pathological changes of the oral mucosa were concurrently observed in a single subject [3], no psoriasis-specific lesions or nonspecific lesions of the oral mucosa were reported on dental charts. However, in the study by Olejnik et al. [3], clinically healthy oral mucosa was observed in 31% of psoriasis patients. The limited occurrence of oral psoriasis has been generally attributed to variations in the expression of surface carbohydrates between oral and cutaneous tissues. For instance, the glycoprotein corneodesmosin, believed to play a role in psoriasis development, is present in cutaneous epithelia but not in mucosal epithelia [15].

Acosta Felquer et al. proposed, on the other hand, that the actual prevalence of oral psoriasis may be underestimated because the assessment of the oral mucosa is typically not a standard component of a dermatological examination in psoriasis, thus, underlying the importance of thorough examination of oral mucosa in psoriasis patients [10]. However, current participants had undergone a complete oral examination reported in dental charts. Consequently, a more likely explanation for our results may rely on the evidence that oral findings often exhibit transient, migratory characteristics, with daily fluctuations corresponding to the exacerbation or remission of cutaneous lesions [15].

Furthermore, oral lesions in psoriasis patients are thought to be more frequent in association with specific subtypes of psoriasis, such as the pustular (nine cases) or erythrodermic variants (two cases) [15], which were rarely or never detected in the present chart review.

Contrary to our results, Manzano et al. [25] also reported a higher frequency of oral lesions and abnormal oral mucosa in subjects with rheumatoid arthritis and systemic lupus erythematosus, although 10% of the presently investigated subjects suffered from other IMIDs.

#### 4.1.2. Oral Health-Related Quality of Life (OHRQoL) in Adult Untreated Psoriasis Subjects

Drawing from the World Health Organization’s [WHO] International Classification of Impairments, Disabilities, and Handicaps (ICIDH) framework [30], Locker’s conceptual model of oral health [31] established links between oral disorders and their biological, behavioral, and psychosocial consequences. Indeed, there is a growing acknowledgment that oral disorders can significantly affect physical, social, and psychological well-being [32]. Therefore, it is reasonable to assume that a deterioration in Oral Health-Related Quality of Life (OHRQoL) due to illness, as the oral complications associated with numerous systemic diseases, can potentially compromise overall Quality of Life [17]. This realization has led to an increasing focus on improving quality of life as the primary outcome of dental treatment. Accordingly, several instruments have been developed to measure dental outcomes by assessing the impact of changes in oral health on quality of life [32].

The original OHIP questionnaire, assessing the impact of oral health status on quality of life, was formulated and validated by Slade and Spencer [33]. It comprised 49 questions and measured seven dimensions, including functional limitations, physical pain, psychological distress, physical disability, psychological disability, social disability, and other disabilities, and had well-documented psychometric properties [24,34].

However, since its introduction, the abbreviated OHIP-14 has been preferred by many researchers over the OHIP-49 because it is more practical [32], similar to the present study. The use of the OHIP-14 short form aimed to reduce the burden on patients and clinicians [17], which favored the current completion rate of 100% and is consistent with other reported results, with less than 1% of responses containing missing items, demonstrating good patient compliance [17]. The completion rate may have been positively influenced by the fact that the questionnaire was conducted as an interview, as in other studies, to overcome the diversity of the sample [17,35,36].

The OHIP-14 scores currently recorded ranged from minimum to maximum values (0–56), with nearly 75% of psoriasis patients revealing an excellent OHRQoL (OHIP score 0–14), 20% having good OHRQoL (OHIP score 15–28), and approximately 3% and 2% declaring moderate OHRQoL (OHIP score 27–42) and low OHRQoL (OHIP score 43–56), respectively. These results are much more favorable than those of Olejnik et al. [3], who examined the oral health status and dental treatment needs of psoriasis patients under different therapeutic regimens in managing psoriasis and found that only about 10% of participants did not require dental intervention. In addition, these Authors reported a higher need for treatment associated with topical therapy for psoriasis [3]. Therefore, our largely more favorable results may be partially because the current eligibility criteria only consider individuals with psoriasis who are not on treatment. One of the purposes of this exclusion criterion was to avoid misdiagnosis of oral lesions and conditions attributable or linked to the pharmacologic treatment of psoriasis and its associated potential side effects.

Apart from the psoriasis patients, our predominantly positive OHIP-14 scores were superior even compared to the general population, possibly because the data from subjects with untreated dental infections were also not considered. In fact, tooth decay proved to be the oral condition that exerted the most significant impact on OHQRoL, even beyond edentulism [17,37]. In any case, despite having at least 15 teeth, nearly 95% of participants reported excellent/good OHRQoL, whereas poorer OHRQoL had previously been observed in subjects with <20 teeth [17].

In contrast, the exclusion of psoriasis patients with active periodontal infections and removable denture wearers should have had no effect on the OHIP-14 scores presented, as periodontal pockets ≥ 4 mm and denture status showed little effect on OHRQoL [17].

Furthermore, it could be suggested that our positive results are because the dental charts examined were from individuals who, unlike most studies, were not necessarily seeking treatment for existing oral problems [17]. Indeed, individuals who felt the need for dental treatment generally had poorer OHRQoL, indicating their ability to understand and assess their symptoms [17]. Consequently, symptoms, the number of teeth, and pain strongly correlated with increased OHIP-14 scores and worse OHRQoL [32]. In turn, it has been suggested that selecting patients based on a higher or lower baseline level of a particular variable may lead to regression to the mean bias, which is influenced by the biological variability of the data or errors [32].

Our findings differ from Nuttall et al.’s model hierarchy, which identified experiencing a handicap as the least frequent impact [37].

### 4.2. Correlation of DLQI and OHIP-14 Scores with Sample and Psoriasis Descriptive Variables in Adult Untreated Psoriasis Subjects

Skin diseases, including psoriasis, have been demonstrated to significantly impact patients’ health-related quality of life (HRQoL) with increased DLQI scores [38]. While psoriasis generally does not impact survival, it significantly diminishes the quality of life for affected individuals, as evidenced by the substantial detriment reported in various studies [39]. Indeed, psoriasis can affect various facets of patients’ lives, encompassing their careers, incomes, relationships, and physical intimacy [38].

Although in the present chart review, the mean duration of the disease was 16.2 years (median of 11.0 years; standard deviation of 13.8 years), similar to that (18 years) described by Olejnik et al. [3], no correlation was presently found between DLQI scores and the duration since psoriasis diagnosis. Nevertheless, determining the precise age of onset and/or diagnosis poses challenges, as studies typically rely on patient recall or the physician’s recorded diagnosis during the initial visit. Relying on patient recall, such as in the present study, can introduce inaccuracies, while basing onset on the first physician visit might underestimate disease occurrence, given potential years of minimal symptoms before seeking consultation [11,40]. In any case, the improvement in DLQI scores with age could be linked to a sort of adaptation and lower expectations of older psoriasis patients.

Conversely, DLQI scores displayed positive significant associations with both PASI and BSA (Table 1) scores, as expected. In fact, it is well known that the severity of psoriasis can increase, and moderate-to-severe cases may substantially compromise patients’ overall quality of life (QoL) [41]. Moreover, psoriasis can lead to diverse stressful experiences for patients [42]. Patients with psoriasis commonly feel stigmatized by their condition, contributing to daily disability, depression, and suicidal ideation in more than 5% of patients [11,43]. Existing literature reviews have outlined numerous challenges in coping with this chronic disease, spanning issues related to treatment management, symptom control, management of assaults on body image and self-esteem, and the ability to lead a normal life. Patients vary in their adjustment to chronic illness, with those with psoriasis reporting various psychosocial consequences such as social isolation and feelings of anger, depression, shame, and anxiety [44].

The impairment of quality of life in psoriasis patients has been emphasized in the work of Finlay, with reductions similar to or worse than those experienced by patients with other chronic diseases such as ischaemic heart disease and diabetes [11,45]. Accordingly, it is not surprising that OHIP-14 scores displayed positive correlations with a medical history of hypertension and IMID (Table 2). In any case, OHIP-14 values (mean: 8.58, median: 3, standard deviation: 11.6) may not have been excessively affected because approximately half (43/90 subjects) of the participants had not reported comorbidities or habitual drug intake.

Contrary to observations by Khalifa et al., no correlation was found between OHRQoL (OHIP-14 scores) and psoriasis patients’ age. Even so, our finding is in agreement with multiple previous observations [17,46,47], reporting that adults aged 70 years or older generally had better mean impact scores compared to younger age groups, possibly due to lower expectations for oral health in older adults. A possible explanation may rely on the evidence that psoriasis is known to manifest at any age, with reported cases from birth to advanced older individuals [3], although several large studies have identified a bimodal age of onset [11,40]. The mean age of initial psoriasis presentation ranges from 15 to 20 years, with a second peak observed at 55–60 years [11,40]. In the present study, participants were between 18 and 77 years of age with a mean of 52.1 and a median of 53.0 (standard deviation: 14.1), and their self-perception of oral health and the quality of life associated with it could, therefore, be similar to that of older subjects.

### 4.3. Comparison of Sample Characteristics, Psoriasis Descriptive Variables by DLQI and OHQRoL

The differences between men and women in OHQRoL were not statistically significant in either age classes. Our results contrast with previous observations that women generally have poorer OHRQoL than men on OHIP-14 measures, hypothesized because women are generally considered more stress-prone [17] and stress is likely to have a stronger impact on the psychological aspects of HRQoL in women compared to men [48]. Indeed, stress is described by up to 60% of patients as an exacerbator or trigger of their disease and is known to regulate the immune response, whereby an abnormal neuroendocrine response to stress may contribute to the pathogenesis of chronic autoimmune diseases such as rheumatoid arthritis [11,49]. Further research is needed to understand the possible abnormal response of the hypothalamic–adrenal axis to acute stress in some patients with psoriasis [11]. Recent research shows that pathological worry and anxiety are prevalent in a significant proportion of psoriasis patients, with psychological and interpersonal difficulties affecting various aspects of daily life [11,50]. In addition, previous research has shown that women are more likely to react to stress and show more discomfort and stigmatization than men [48].

However, it has been suggested that individuals experience the effects of their oral condition differently, with some facing relevant consequences [17]. Indeed, several non-clinical patient characteristics may contribute significantly to the unexplained variation in OHIP-14 levels [32]. In addition, OHIP showed the strongest correlation with negative affect, which is defined as a general predisposition to experience distress and includes aversive mood states such as disgust, anger, guilt, contempt, anxiety, and depression. The ‘symptom perception hypothesis’ states that individuals with a particular personality trait, such as negative affect, are likelier to perceive and complain about health problems. A deeper understanding of the psychological factors that influence OHRQoL could have implications for assessing the need for dental care and determining appropriate gender-specific interventions.

PASI nd BSA were significantly more pronounced in participants with DLQI > 10 compared to DLQI ≤ 10, as expected. Conventional methods of assessing psoriasis severity include determining the area affected about the total body surface (body surface area, BSA), using the Psoriasis Area and Severity Index (PASI) to assess lesions based on erythema, induration, scaling and area affected, and incorporating the physician’s global assessment (PGA) for an overarching assessment of lesion severity [7]. In Europe, the PASI is a standard tool for grading the severity of psoriasis and is often used as a primary or secondary endpoint in international clinical trials [7]. Despite certain methodological limitations, this scoring system is particularly beneficial in patients with moderate to severe psoriasis and a reliable tool for assessing treatment success or failure when patients are assessed before treatment initiation and during therapy [51], as in the present study. The reliability of the PASI and the high percentage (approximately 80%) of participants currently diagnosed with moderate to severe psoriasis can undoubtedly support our findings.

Additionally, diffuse lesion distribution, currently found in only nine subjects, correlated significantly with HRQoL if involving the exposed areas [52]. Visibility is linked to self-esteem, as appearance plays a central role in social and cultural environments [52]. Consequently, chronic dermatological diseases have a significant impact on patients’ mental health, self-esteem, and body image. Especially in the case of psoriasis on the neck, patients often experience great anxiety [48]. In this regard, the PASI, which only considers the regional body surface area (BSA), does not take into account the disproportionate burden on visible/sensitive areas [42,48]. Therefore, clinicians must consider the impact of serious illness on various aspects of patients’ lives, including endeavoring to address this issue. Recent literature has emphasized the role of psychological factors in the incidence of relapse and remission, as well as in treatment dynamics [53], such that improving quality of life emerges as an essential treatment goal for patients with psoriasis [48]. This attention is justified by the importance of the skin for aesthetics and appearance, its role in non-verbal communication, and its involvement in emotional expression.

Moreover, although nearly 6–11% of individuals with psoriasis may experience inflammatory arthropathy, commonly known as psoriasis arthritis [54], 23.4% of participants suffered from psoriatic arthritis, while 15.3% had a prevailing mucosal (mainly genital) involvement.

Significantly, in our sample, significant differences in OHQRoL between individuals with and without comorbidities. In detail, among subjects revealing an Excellent OHQRoL (OHIP score of 0–14), 92.6% were non-IMID (*p*-value = 0.02), 75% were non-hypertensive (*p*-value = 0.00), 89.7% were non-diabetic (*p*-value = 0.02), and 86.8% were non-CVD (*p*-value = 0.01). Accordingly, given that psoriasis extends beyond the skin or joints and induces a chronic systemic inflammatory state, it has generally been considered a potential contributor to hypertension, diabetes, dyslipidemia, immune-mediated inflammatory diseases (IMID), and cardiovascular events [55,56,57,58]. Indeed, numerous studies have reported a higher prevalence of these comorbidities in subjects with psoriasis, encompassing both mild and severe cases [55,56,57,58]. Specifically, the correlation between psoriasis and hypertension might be grounded in shared pathways such as the altered renin-angiotensin system, endothelial dysfunction, and increased oxidative stress [58]. The link between psoriasis and diabetes could, in part, be explained by the rise in obesity and unhealthy lifestyles, the insulin resistance associated with inflammation, and the presence of various genes (CDKAL1, PTPN22, ST6GAL1, JAZF1) linked to both conditions [59,60]. Concerning dyslipidemia, Mehta et al. [61] found altered HDL efflux capability in psoriasis subjects and altered lipoproteins A and B levels compared to the healthy controls [62]. Flammer and Ruschitzka [62] introduced the “two plaques for one syndrome” hypothesis, suggesting that molecular pathways and pro-inflammatory cytokines common to both psoriasis and atherosclerosis contribute to a similar inflammatory infiltrate of T cells, macrophages, and monocytes.

Moreover, Rebelo et al. [63] explored the relationship between OHRQoL in adults with systemic arterial hypertension and periodontal status, revealing a synergistic effect likely based on shared risk factors between the two conditions. Vu et al. [64] demonstrated that diabetic individuals with untreated dental or periodontal infections and smoking habits had lower OHRQoL. Nevertheless, in our study, significant differences were identified in subjects with both diabetes and psoriasis compared to those solely experiencing psoriasis, although subjects with untreated dental or periodontal infections and smoking habits, factors associated with lower OHRQoL scores in the study by Vu et al. [64].

Based on the outcomes of both the previous and present studies, the coexistence of comorbidity and psoriasis does not appear to significantly influence OHRQoL scores compared to the isolated presence of either one comorbidity or psoriasis alone.

From a research design perspective, the current study lacks the robustness to prove causal conclusions. Moreover, it is crucial to acknowledge that the predominantly positive OHIP-14 scores indicating favorable OHRQoL associated with psoriasis may be overestimated. This potential overestimation results from the exclusion criteria, which include the exclusion of patients with fewer than 15 teeth, smokers, and people with removable dentures. These exclusions could lead to biases that affect both oral health status and subjective perceptions of health status among study participants.

As a counterpart, despite the extensive literature addressing the impact of various health conditions on OHRQoL, our study is the first to examine the specific impact of psoriasis on OHRQoL using the OHIP-14 questionnaire to shed light on the intricate relationship between psoriasis and health-related quality of life (HRQoL). The deliberate decision not to use online questionnaires in our study adds strength by mitigating the potential bias associated with internet access and computer literacy.

In addition, although reduced in size also due to the restricted eligibility criteria, similar to a previous study in South Korea [48], our sample reflects the general population and maintains a representative male-to-female ratio among psoriasis patients, increasing the generalizability of the results of our study.

#### Furthermore, Oral Lesions

Future studies should consider a more extensive and more diverse sample to improve the generalizability of the results and gain insight into the different effects of psoriasis on oral health in different population groups.

In addition, future research should evaluate the associations between oral health status and psoriasis in patients undergoing treatment so that dentists and dermatologists can focus their attention on the oral condition and its impact on the OHRQoL of these individuals with greater physical-emotional vulnerability.

## 5. Conclusions

The present study retrospectively reviewed the prevalence of specific and nonspecific oral lesions and Oral Health-Related Quality of Life (OHRQoL), self-assessed using the Oral Health Impact Profile-14 (OHIP-14) questionnaire, in 90 adult untreated psoriasis patients with ≥15 teeth, no smoking habits, and no dental or periodontal infections.

No oral lesions were detected and excellent/good OHRQoL was found in 94% of cases. As expected, DLQI correlated positively with PASI and BSA and negatively with duration since psoriasis diagnosis, and OHIP-14 correlated positively with certain comorbidities (hypertension, type 2 diabetes, IMID, and cardiovascular events). Prevailing skin involvement was higher in men and in subjects within 10 years since psoriasis diagnosis, while diffuse lesions distribution over 10 years since diagnosis.

Future studies should investigate the long-term effects of psoriasis, the impact of psoriasis treatment, and the underlying biological mechanisms to elucidate the full relationship between psoriasis and oral health outcomes.

## Figures and Tables

**Table 1:** Linear regression models for DLQI scores using as principal covariates PASI and BSA: (**A**) the covariates for adjusting in the first model were age, gender, and years since psoriasis diagnosis; (**B**) the covariates for adjusting in the second model were all the comorbidities investigated; (**C**) the covariates for adjusting in the third model were the psoriatic lesions variables.

DLQI			
A			
Characteristic	Beta	95% CI ^1^	*p*-Value
(Intercept)	8.873	1.902, 15.844	0.013
PASI	0.362	0.146, 0.578	0.001 *
BSA	0.105	−0.015, 0.226	0.085
Age	−0.002	−0.118, 0.115	0.97
Gender			
Female	—	—	
Male	−0.691	−3.930, 2.548	0.67
Years since diagnosis	−0.080	−0.203, 0.042	0.19
**B**			
**Characteristic**	**Beta**	**95% CI** ^1^	***p*** **-Value**
(Intercept)	7.041	3.926, 10.155	<0.001
PASI	0.274	0.055, 0.493	0.015 *
BSA	0.132	0.018, 0.246	0.023 *
IMID			
No	—	—	
Yes	−3.520	−9.219, 2.179	0.22
Hypertension			
No	—	—	
Yes	−0.345	−4.046, 3.356	0.85
Dyslipidemia			
No	—	—	
Yes	1.326	−2.780, 5.431	0.52
Diabetes			
No	—	—	
Yes	−1.667	−6.734, 3.400	0.51
CVD			
No	—	—	
Yes	2.894	−1.762, 7.550	0.22
**C**			
**Characteristic**	**Beta**	**95% CI** ^1^	***p*** **-Value**
(Intercept)	7.491	3.674, 11.308	<0.001
PASI	0.376	0.156, 0.597	0.001 *
BSA	0.130	0.012, 0.248	0.032 *
Skin Involvement			
No	—	—	
Yes	−0.502	−4.351, 3.347	0.80
Nail Involvement			
No	—	—	
Yes	−2.028	−6.389, 2.333	0.36
Mucosal Involvement			
No	—	—	
Yes	−1.070	−5.391, 3.252	0.62
Diffuse Lesions			
No	—	—	
Yes	−2.530	−8.565, 3.505	0.41

^1^ CI = Confidence Interval; * *p* < 0.05; CVD = Cardiovascular disease.

**Table 2:** Linear regression models for OHIP-14 scores using as principal covariates PASI and BSA: (**A**) the covariates for adjusting in the first model were age, gender, and years since psoriasis diagnosis; (**B**) the covariates for adjusting in the second model were all the comorbidities investigated; (**C**) the covariates for adjusting in the third model were the psoriatic lesions variables.

OHIP			
A			
Characteristic	Beta	95% CI ^1^	*p*-Value
(Intercept)	−10.467	−21.110, 0.177	0.054
PASI	0.027	−0.303, 0.357	0.87
BSA	0.081	−0.102, 0.265	0.38
Age	0.318	0.140, 0.496	<0.001 *
Gender			
Female	—	—	
Male	1.819	−3.127, 6.765	0.47
Years since diagnosis	−0.031	−0.218, 0.155	0.74
**B**			
**Characteristic**	**Beta**	**95% CI** ^1^	***p*** **-Value**
(Intercept)	1.238	−3.180, 5.656	0.58
PASI	0.213	−0.098, 0.523	0.18
BSA	0.053	−0.109, 0.215	0.52
IMID			
No	—	—	
Yes	11.182	3.098, 19.265	0.007 *
Hypertension			
No	—	—	
Yes	7.722	2.472, 12.971	0.004 *
Dyslipidemia			
No	—	—	
Yes	−0.743	−6.567, 5.080	0.80
Diabetes			
No	—	—	
Yes	4.572	−2.615, 11.758	0.21
CVD			
No	—	—	
Yes	1.584	−5.020, 8.188	0.63
**C**			
**Characteristic**	**Beta**	**95% CI ^1^**	***p*** **-Value**
(Intercept)	8.084	2.039, 14.129	0.009
PASI	0.044	−0.306, 0.393	0.80
BSA	0.027	−0.160, 0.214	0.77
Skin Involvement			
No	—	—	
Yes	−3.493	−9.588, 2.602	0.26
Nail Involvement			
No	—	—	
Yes	1.828	−5.078, 8.734	0.60
Mucosal Involvement			
No	—	—	
Yes	7.257	0.413, 14.101	0.038 *
Diffuse Lesions			
No	—	—	
Yes	3.464	−6.093, 13.021	0.47

^1^ CI = Confidence Interval; * *p* < 0.05; CVD = Cardiovascular disease.

**Table 3:** Differences in baseline characteristics of DLQI classes tested by t-student or Wilcoxon tests (according to their distribution) for continuous variables, and by Pearson chi-squared or Fisher’s exact tests for categorical variables.

	Dermatology Life Quality Index		
Characteristic	DLQI ≤ 10, N = 41 ^1^	DLQI > 10, N = 48 ^1^	*p*-Value ^2^
Age	53.00 (13.00)	53.00 (14.75)	0.69
Gender			0.40
Female	20.0 (48.8%)	19.0 (39.6%)	
Male	21.0 (51.2%)	29.0 (60.4%)	
Years Diagnosis	15.00 (24.00)	10.00 (15.00)	0.20
PASI	3.00 (5.00)	11.00 (9.00)	0.00 *
BSA	15.00 (23.00)	30.00 (20.00)	0.00 *
IMID			0.29
No	35.0 (85.4%)	45.0 (93.8%)	
Yes	6.0 (14.6%)	3.0 (6.2%)	
Hypertension			0.51
No	25.0 (61.0%)	33.0 (68.8%)	
Yes	16.0 (39.0%)	15.0 (31.2%)	
Dyslipidemia			0.29
No	35.0 (85.4%)	36.0 (75.0%)	
Yes	6.0 (14.6%)	12.0 (25.0%)	
Diabetes (Type 2)			1.00
No	35.0 (85.4%)	42.0 (87.5%)	
Yes	6.0 (14.6%)	6.0 (12.5%)	
Cardiovascular disease			0.27
No	36.0 (87.8%)	37.0 (77.1%)	
Yes	5.0 (12.2%)	11.0 (22.9%)	
Skin involvement			0.38
No	17.0 (41.5%)	15.0 (31.2%)	
Yes	24.0 (58.5%)	33.0 (68.8%)	
Nail involvement			0.42
No	35.0 (85.4%)	37.0 (77.1%)	
Yes	6.0 (14.6%)	11.0 (22.9%)	
Mucosal involvement			0.27
No	36.0 (87.8%)	37.0 (77.1%)	
Yes	5.0 (12.2%)	11.0 (22.9%)	
Diffuse lesions			0.73
No	36.0 (87.8%)	44.0 (91.7%)	
Yes	5.0 (12.2%)	4.0 (8.3%)	

^1^ Median (IQR) or Frequency (%); ^2^ Wilcoxon rank sum test; Fisher’s exact test; * statistically significant.

**Table 4:** Differences in baseline characteristics of OHRQoL classes tested by ANOVA or Kruskal–Wallis tests (according to their distribution) for continuous, and Pearson chi-squared or Fisher’s exact tests for categorical variables.

	Oral Health-Related Quality of Life				
Characteristic	Low, N = 2 ^1^	Medium, N = 5 ^1^	Good, N = 15 ^1^	Excellent, N = 68 ^1^	*p*-Value ^2^
Age	65.50 (9.50)	69.00 (4.00)	53.00 (19.50)	52.00 (13.75)	0.00 *
Gender					0.51
Female	0.0 (0.0%)	1.0 (20.0%)	7.0 (46.7%)	32.0 (47.1%)	
Male	2.0 (100.0%)	4.0 (80.0%)	8.0 (53.3%)	36.0 (52.9%)	
Years Diagnosis	8.00 (0.00)	20.00 (10.00)	12.00 (29.50)	11.00 (14.25)	0.90
PASI	1.00 (1.00)	16.00 (20.00)	4.00 (7.50)	7.00 (9.00)	0.01 *
BSA	17.50 (17.50)	40.00 (25.00)	17.00 (24.00)	25.00 (25.00)	0.11
IMID					0.02 *
No	0.0 (0.0%)	5.0 (100.0%)	13.0 (86.7%)	63.0 (92.6%)	
Yes	2.0 (100.0%)	0.0 (0.0%)	2.0 (13.3%)	5.0 (7.4%)	
Hypertension					0.00 *
No	0.0 (0.0%)	1.0 (20.0%)	7.0 (46.7%)	51.0 (75.0%)	
Yes	2.0 (100.0%)	4.0 (80.0%)	8.0 (53.3%)	17.0 (25.0%)	
Dyslipidemia					0.13
No	2.0 (100.0%)	2.0 (40.0%)	13.0 (86.7%)	55.0 (80.9%)	
Yes	0.0 (0.0%)	3.0 (60.0%)	2.0 (13.3%)	13.0 (19.1%)	
Diabetes (Type 2)					0.02 *
No	0.0 (0.0%)	5.0 (100.0%)	12.0 (80.0%)	61.0 (89.7%)	
Yes	2.0 (100.0%)	0.0 (0.0%)	3.0 (20.0%)	7.0 (10.3%)	
Cardiovascular disease					0.01 *
No	2.0 (100.0%)	1.0 (20.0%)	12.0 (80.0%)	59.0 (86.8%)	
Yes	0.0 (0.0%)	4.0 (80.0%)	3.0 (20.0%)	9.0 (13.2%)	
Skin involvement					0.19
No	0.0 (0.0%)	3.0 (60.0%)	8.0 (53.3%)	21.0 (30.9%)	
Yes	2.0 (100.0%)	2.0 (40.0%)	7.0 (46.7%)	47.0 (69.1%)	
Nail involvement					0.14
No	2.0 (100.0%)	5.0 (100.0%)	9.0 (60.0%)	57.0 (83.8%)	
Yes	0.0 (0.0%)	0.0 (0.0%)	6.0 (40.0%)	11.0 (16.2%)	
Mucosal involvement					0.03 *
No	0.0 (0.0%)	3.0 (60.0%)	13.0 (86.7%)	57.0 (83.8%)	
Yes	2.0 (100.0%)	2.0 (40.0%)	2.0 (13.3%)	11.0 (16.2%)	
Diffuse lesions					0.13
No	2.0 (100.0%)	3.0 (60.0%)	13.0 (86.7%)	63.0 (92.6%)	
Yes	0.0 (0.0%)	2.0 (40.0%)	2.0 (13.3%)	5.0 (7.4%)	

^1^ Median (IQR) or Frequency (%); ^2^ Kruskal–Wallis rank sum test; Fisher’s exact test; * Statistically significant.

## Data Availability

Data supporting reported results can be found in Web Of Science, Scopus, and MEDLINE/PubMed databases.

## Supplementary Materials

The following supporting information can be downloaded at: https://www.mdpi.com/article/10.3390/life14030347/s1, File S1: Oral Health Impact Profile (OHIP-14)—Italian version.
