# Supplementary material for: Oral Lesions and Oral Health-Related Quality of Life in Adult Patients with Psoriasis: A Retrospective Chart Review
Source: Life (Basel). 2024 Mar 7;14(3):347. doi: 10.3390/life14030347 (PMC10971651; doi:10.3390/life14030347)
Supplement: Supplementary file 1 [file life-14-00347-s001.zip › life-2871708-supplementary.pdf]

Supplementary file 1 (S1) - Oral Health Impact Profile (OHIP-14) – Italian version.

The Italian version of the questionnaire is based on a self-reported evaluation of the oral health-related quality of life through 14 questions (Oral Health Impact Profile – OHIP-14). The answers were based on a five-level rating scale.

1. **HA AVUTO PROBLEMI NEL PRONUNCIARE PAROLE A CAUSA DI PROBLEMI CON DENTI, BOCCA, O PROTESI?**

   ⎕ **mai**(=0) ⎕ **quasi mai** (=1) ⎕ **qualche volta** (=2) ⎕ **abbastanza spesso** (=3) ⎕ **molto spesso** (=4)

2. **HA TROVATO CHE IL SUO SENSO DEL GUSTO FOSSE PEGGIORATO A CAUSA DI PROBLEMI CON DENTI, BOCCA, O PROTESI?**

   ⎕ **mai**(=0) ⎕ **quasi mai** (=1) ⎕ **qualche volta** (=2) ⎕ **abbastanza spesso** (=3) ⎕ **molto spesso** (=4)

3. **HA AVUTO DOLORI FASTIDIOSI IN BOCCA?**

   ⎕ **mai**(=0) ⎕ **quasi mai** (=1) ⎕ **qualche volta** (=2) ⎕ **abbastanza spesso** (=3) ⎕ **molto spesso** (=4)

4. **HA TROVATO DIFFICILE MANGIARE ALCUNI CIBI A CAUSA DI PROBLEMI CON DENTI, BOCCA, O PROTESI?**

   ⎕ **mai**(=0) ⎕ **quasi mai** (=1) ⎕ **qualche volta** (=2) ⎕ **abbastanza spesso** (=3) ⎕ **molto spesso** (=4)

5. **SI È SENTITO A DISAGIO DI FRONTE AGLI ALTRI A CAUSA DI PROBLEMI CON DENTI, BOCCA, O PROTESI?**

   ⎕ **mai**(=0) ⎕ **quasi mai** (=1) ⎕ **qualche volta** (=2) ⎕ **abbastanza spesso** (=3) ⎕ **molto spesso** (=4)

6. **SI È SENTITO TESO O NERVOSO A CAUSA DI PROBLEMI CON DENTI, BOCCA, O PROTESI?**

   ⎕ **mai**(=0) ⎕ **quasi mai** (=1) ⎕ **qualche volta** (=2) ⎕ **abbastanza spesso** (=3) ⎕ **molto spesso** (=4)

7. **LA SUA DIETA È STATA INSODDISFACENTE A CAUSA DI PROBLEMI CON DENTI, BOCCA, O PROTESI?**

   ⎕ **mai**(=0) ⎕ **quasi mai** (=1) ⎕ **qualche volta** (=2) ⎕ **abbastanza spesso** (=3) ⎕ **molto spesso** (=4)

8. **HA DOVUTO INTERROMPERE I PASTI A CAUSA DI PROBLEMI CON DENTI, BOCCA, O PROTESI?**

   ⎕ **mai**(=0) ⎕ **quasi mai** (=1) ⎕ **qualche volta** (=2) ⎕ **abbastanza spesso** (=3) ⎕ **molto spesso** (=4)

9. **HA TROVATO DIFFICOLTÀ A RILASSARSI A CAUSA DI PROBLEMI CON DENTI, BOCCA, O PROTESI?**

☐ **mai**(=0) ☐ **quasi mai** (=1) ☐ **qualche volta** (=2) ☐ **abbastanza spesso** (=3) ☐ **molto spesso** (=4)

10. **SI È SENTITO UN PO'IMBARAZZATO A CAUSA DI PROBLEMI CON DENTI, BOCCA, O PROTESI?**

☐ **mai**(=0) ☐ **quasi mai** (=1) ☐ **qualche volta** (=2) ☐ **abbastanza spesso** (=3) ☐ **molto spesso** (=4)

11. **SI È SENTITO UN PO' IRRITABILE CON ALTRE PERSONE A CAUSA DI PROBLEMI CON DENTI, BOCCA, O PROTESI?**

☐ **mai**(=0) ☐ **quasi mai** (=1) ☐ **qualche volta** (=2) ☐ **abbastanza spesso** (=3) ☐ **molto spesso** (=4)

12. **HA AVUTO DIFFICOLTÀ A FARE I LAVORI USUALI A CAUSA DI PROBLEMI CON DENTI, BOCCA, O PROTESI?**

☐ **mai**(=0) ☐ **quasi mai** (=1) ☐ **qualche volta** (=2) ☐ **abbastanza spesso** (=3) ☐ **molto spesso** (=4)

13. **HA TROVATO CHE LA VITA IN GENERALE FOSSE MENO SODDISFACENTE A CAUSA DI PROBLEMI CON DENTI, BOCCA, O PROTESI?**

☐ **mai**(=0) ☐ **quasi mai** (=1) ☐ **qualche volta** (=2) ☐ **abbastanza spesso** (=3) ☐ **molto spesso** (=4)

14. **È STATO TOTALMENTE INCAPACE DI AGIRE O FARE COSE A CAUSA DI PROBLEMI CON DENTI, BOCCA, O PROTESI?**

☐ **mai**(=0) ☐ **quasi mai** (=1) ☐ **qualche volta** (=2) ☐ **abbastanza spesso** (=3) ☐ **molto spesso** (=4)
